# Systematic investigation of the generation of luminescent emitters in hBN via irradiation engineering

**DOI:** 10.1038/s41598-025-24064-x

**Published:** 2025-11-17

**Authors:** Pooja C. Sindhuraj, José M. Caridad, Corné Koks, Moritz Fischer, Denys I. Miakota, Juan A. Delgado-Notario, Kenji Watanabe, Takashi Taniguchi, Stela Canulescu, Sanshui Xiao, Martijn Wubs, Nicolas Stenger

**Affiliations:** 1 Department of Electrical and Photonic Engineering, Technical University of Denmark, 2800 Kongens Lyngby, Denmark; 2 Departmento de Física Aplicada, Universidad de Salamanca, 37008 Salamanca, Spain; 3 Unidad de Excelencia en Luz y Materia Estructuradas (LUMES), Universidad de Salamanca, 37008 Salamanca, Spain; 4 NanoPhoton – Center for Nanophotonics, Technical University of Denmark, 2800 Kgs. Lyngby, Denmark; 5 Research Center for Electronic and Optical Materials, National Institute for Materials Science, 1-1 Namiki, Tsukuba, 305-0044 Japan; 6 Research Center for Materials Nanoarchitectonics, National Institute for Materials Science, 1-1 Namiki, Tsukuba, 305-0044 Japan

**Keywords:** HBN, Oxygen irradiation, Lattice defects, Organic molecules, Two-dimensional materials, Quantum optics, Single photons and quantum effects, Fluorescence spectroscopy

## Abstract

Hexagonal boron nitride (hBN), a two-dimensional (2D) material, garners interest for hosting bright quantum emitters at room temperature. While crystallographic defects are widely believed to be the source of these emitters, their exact nature, especially for visible frequencies, remains debated. Carbon impurities are frequently implicated, though their precise role is unclear, and extrinsic organic molecules at the hBN-substrate interface have also been proposed as contributors. In this study, we systematically explore the formation of luminescent emitters through irradiation engineering. Our results confirm that low-energy oxygen irradiation followed by annealing is key to forming visible quantum emitters in hBN. Notably, post-annealing in carbon-rich atmospheres significantly increases emitter density, reinforcing carbon’s potential role. We also find that hBN crystallographic quality influences emitter generation, with low-quality hBN producing nearly 20 percent more emitters than high-quality samples. While the formation of extrinsic organic molecules during high-temperature annealing cannot be ruled out, crystallographic defects formed during irradiation are central to emitter creation. We infer that these defects may promote the formation of few-atom luminescent centers and serve as molecular pinning sites. Our systematic study and findings advance the understanding of the formation of visible frequency quantum emitters in hBN.

## Introduction

Quantum emitters (QEs) in hexagonal boron nitride (hBN) have attracted significant interest since their discovery in 2016^1^. These QEs exhibit bright and stable emission, even at room temperature, making them promising for quantum communication, sensing, and metrology applications without the need for costly and power-intensive cryogenic environments^2^. They emit light across a broad range of the electromagnetic spectrum, from near-infrared (NIR) to ultraviolet (UV) frequencies, and some even show magnetic properties at room temperature, which is particularly useful for sensing applications^3–7^.

**Fig. 1:**
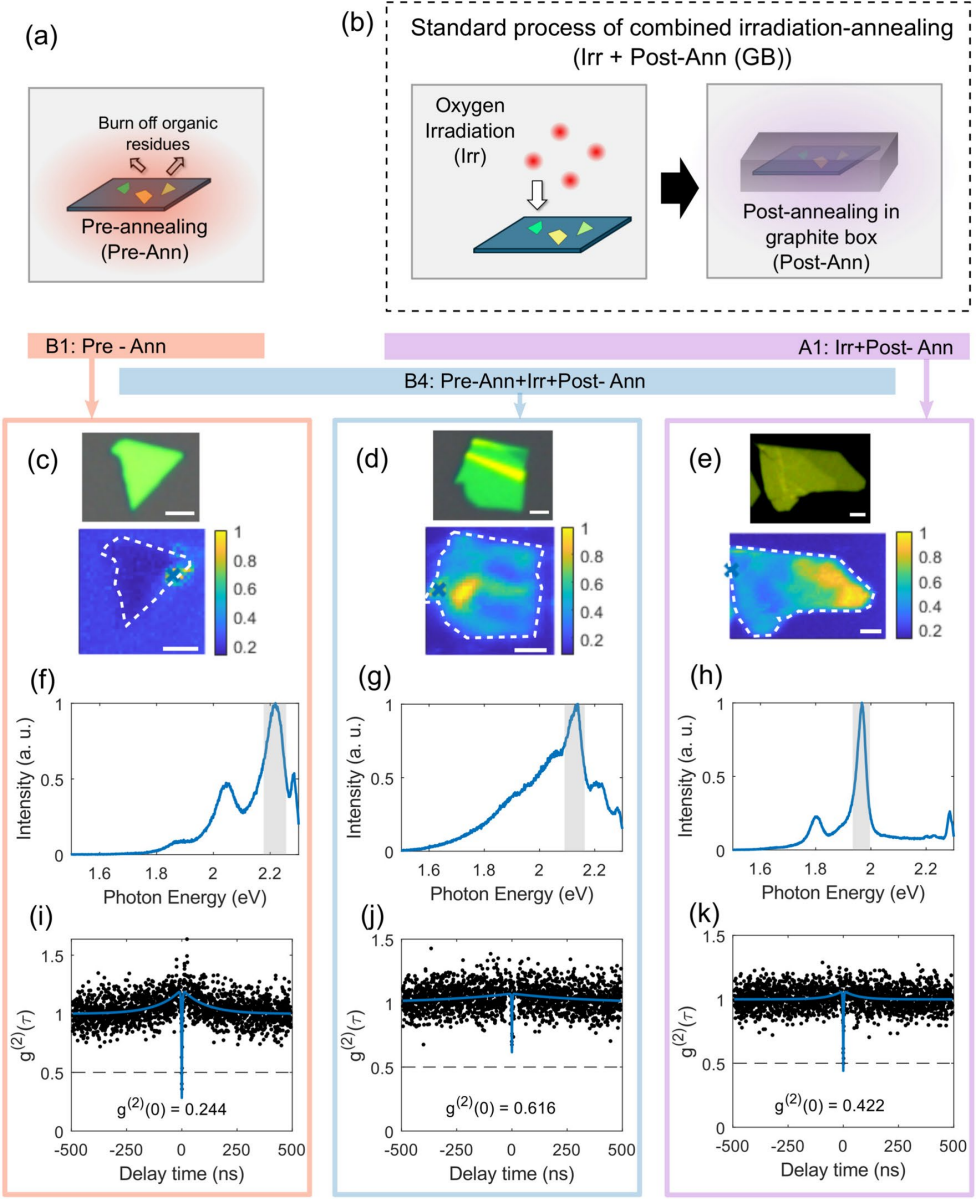
Schematic representation of the (**a**) pre-annealing treatment of exfoliated flakes in oxygen/argon atmosphere done to remove any organic residues, (**b**) standard process involving the oxygen irradiation of exfoliated flakes followed by post-annealing in nitrogen in a graphite box. Optical image (top) and photoluminescence map (bottom) of flakes undergoing (**c**) pre-annealing alone (B1: Pre-Ann), (**d**) pre-annealing followed by the standard process (B4: Pre-Ann+Irr+Post-Ann), (**e**) standard process (A1: Irr+Post-Ann). The white scale bar inside represents 5$\mu$m, and the color bar represents the normalized integrated PL counts. (**f**)–(**h**) Spectrum of a representative emitter generated in the corresponding processes. The location of the emitter is denoted as a blue cross in the PL maps. (**i**)–(**k**) g^(2)^($\tau$) function of the emitters shown in (**f**), (**g**), and (**h**), respectively, from the spectral region shaded in grey. Treatment combinations undergone by different sample batches and the corresponding emitter densities are also listed in Table 1.

A great number of generation mechanisms have been proposed for these quantum emitters including edge creation^8^, focused ion beam irradiation^9–12^, laser irradiation^13,14^, localized strain^15^, bottom-up growth on patterned substrates^16^, and electron beam irradiation^17–19^. All these methods suggest the formation of crystallographic defects in the hBN lattice, analogous to vacancies in diamond^20^ and other bulk semiconductors^21,22^. This leads to the attribution of these emitters to the presence of defects or impurities in the hBN lattice^1,23–26^. In particular the incorporation of carbon impurities has shown to enhance the formation yield of emitters with emission energies around 2 eV in hBN^27,28^. However, the exact microscopic structure of these 2 eV emitters is still missing and many different structures have been proposed such as $\mathrm {V_BC_N\text{-}}$^27^, $\mathrm {V_NC_B}$^28^, $\mathrm {C_2C_N}$^29^, and $\mathrm {C_BC_NC_BC_N}$ ^19^ among many others ^30,31^. Furthermore, to complicate the microscopic origin of the 2 eV emitters even more, recent studies, have also suggested that polycyclic aromatic hydrocarbons (PAHs)^32^ may create bright emitters at the interface between hBN flakes and their supporting substrate when subjecting the samples to an inert annealing step^33^. In line with this observation, other recent works have shown that hBN serves as an excellent substrate for molecular QEs; for example, terrylene molecules adsorbed on hBN flakes showed narrower linewidths and better spectral stability than on other host materials^34–36^. Importantly, many of the aforementioned methods for QEs share common processing steps and, moreover, the presence of natural defects in hBN crystals or residual organic molecules cannot be fully excluded in any of the fabrication approaches^8,9,13,15,19,33^. Unique identifications of the exact microscopic origin of the 2 eV emitters are therefore still beyond state-of-the-art ^30,31,37^, a fact which is further accentuated by the absence of detailed studies reporting the impact of various fabrication parameters.

In this work, we undertake a systematic investigation of the generation mechanism of emitters with zero-phonon line (ZPL) emission energies between 1.6 and 2.3 eV through irradiation with low-energy, high-fluence oxygen atoms, followed by post-annealing steps^28,29,38^. In particular, we exhaustively investigate the role of commonly used parameters such as (i) irradiation energy, (ii) post-annealing with a carbon-rich environment, (iii) pre-annealing with an oxygen-rich environment, and (iv) the type of hBN crystals used. Our study highlights the importance of the irradiation step combined with post-annealing, a widely used method for generating quantum emitters (QEs) in hexagonal boron nitride (hBN)^28,31,38–41^. We show that defects created during irradiation play a central role, either by forming luminescent crystallographic defects or by pinning polycyclic aromatic hydrocarbon (PAH) molecules present in the environment or generated during annealing^33,42^. This work represents an important step toward identifying the microscopic structure of hBN quantum emitters in the 2 eV emission range, a critical requirement for the controlled and on-demand fabrication of quantum emitters. Such advancements are vital for future applications in quantum information processing, sensing, and metrology.

**Table 1:** The table lists the names and combinations of treatments that different batches of our hBN flakes have been subjected to and the recorded emitter densities

Batch	Treatment	Emitter density ($\#/\mu {m}^2$)	
		Type I	Type II
**A1**	**Irr + Post-Ann(GB); Standard process**	**0.0192±0.0019**	-
A2	Irr + Post-Ann(QB)	0.0106±0.0022	-
A3	Post-Ann(GB)	0.0041±0.0015	-
B1	Pre-Ann	0.0093±0.0022	0.0045±0.0010
B2	Pre-Ann + Irr	0.0085±0.0019	0.0046±0.0013
B3	Pre-Ann + Post-Ann(GB)	0.0067±0.0013	0.0118±0.0012
**B4**	**Pre-Ann + Irr + Post-Ann(GB)**	0.0167±0.0019	0.0138±0.0016

All the irradiation processes mentioned in the table are done at $\sim$204 eV.

The following short names are used for different processes, and the specific parameters for the indicated process for a given batch are explained in detail in the Methods and Results sections.

Batches that have undergone the standard process are highlighted in bold.

## Methods

### Sample preparation

We exfoliate hBN flakes from bulk crystals purchased from HQGraphene onto cleaned SiO_2_/Si substrates. The substrate chips are cleaned with 2 min sonication in acetone and 1 min sonication in isopropyl alcohol prior to the exfoliation. Exfoliated samples are irradiated with oxygen within a Reactive Ion Etch Chamber (RIE). The RIE generates highly directional oxygen plasma targeted to the sample at a flow of 40 sccm, maintaining a 25 mTorr pressure inside the chamber. While setting the chamber pressure and gas flow rate constant, the plasma power that can be manually set determines the kinetic energy at which the plasma reaches the sample. For the plasma powers at 20 W, 40 W, and 60 W, kinetic energies at 120 eV, 204 eV, and 280 eV are achieved, respectively, and are reproducible with an error bar of ±5 eV. The RIE reactor is driven by a high-frequency generator operating at 13.56 MHz and is capacitively coupled to the bottom electrode. The chamber has a circular parallel plate geometry with a plate diameter of 33.5 cm (plate area $\approx 882$ cm$^2$), enabling thus the calculation of the power density of each process. Similar to other commercial tools and studies in literature^43–46^, our RIE reactor does not include a sensor to measure the ion current density. However, we can roughly estimate this quantity with the given process parameters (see supplementary material, Sect. S1). The irradiation is done for 10 seconds in all cases and at room temperature. We chose 10 s to avoid the soot formation as evidenced by the Raman spectra (see the supplementary material, Sect. S2). Following the irradiation, final activation annealing (post-annealing) step is done in a tube furnace in nitrogen at a pressure of 80 mbar without a flow, at a temperature of 850 °C for 30 min. The samples are placed in a graphite box (GB, unless stated otherwise) inside the furnace to ensure the presence of carbon in the environment. This is the so-called standard process^28^ we use for emitter generation as schematically represented in Fig. 1b. Indicated batches undergo post-annealing in a quartz boat (QB).

Some batches of samples undergo an additional annealing step after the exfoliation, prior to any oxygen irradiation or post-annealing steps (see Fig.  1a). This so-called ‘pre-annealing’ process is done in an argon environment with 10$\%$ (v/v) oxygen at a flow rate of 300 sccm, while maintaining a pressure of approximately 0.7 atm, at a temperature of 700°C. The temperature ramp rate for heating and cooling is set at 40°C/min, with the entire process spanning roughly two hours. This annealing step is meant to remove organic contaminants on or around the hBN flakes^33^.

In Sects. 3.1, and 3.2, we show results for bulk hBN crystals purchased from the commercial supplier HQGraphene, referred to as ‘Type I’ samples. We also studied hBN flakes exfoliated from crystals synthesized in a high-pressure, high-temperature process (HPHT) from the National Institute for Materials Science (NIMS)^47,48^ in experiments involving oxygen pre-annealing, referred to as ‘Type II’ samples in this work. In Sect. 3.3, we distinguish the generated emitter densities between these two types of hBN crystals for various processing methods involving oxygen pre-annealing. The batch names, the treatments undergone by each batch, and the corresponding emitter densities are also listed in Table 1.

### Optical characterization

For the photoluminescence measurements, a laser at 516 nm excites the samples, and a 50X microscope objective (0.6 NA) collects the emitted light from the samples filtered by a long-pass filter at 532 nm to the spectrometer or to the single-photon detectors during the intensity correlation measurements. All measurements are performed at room temperature. The calculation of the areal number density of emitters is discussed in detail in the supplementary material, Sect. S3.

**Fig. 2:**
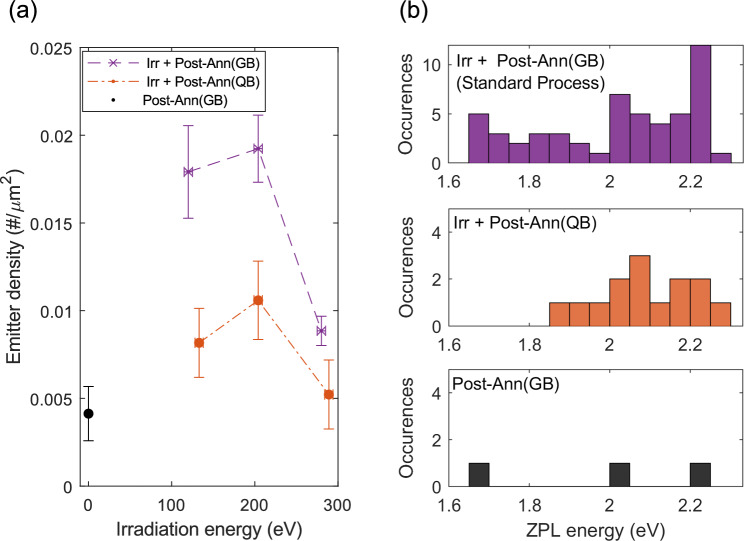
(**a**) Density of emitters plotted against the irradiation energy for the three different processes as indicated. Irr + Post-Ann(GB), shown in purple markers, indicates the standard process in which the samples undergo irradiation at indicated energies at room temperature, followed by nitrogen annealing in a graphite box (A1). Irr + Post-Ann(QB) shown in orange markers indicates the irradiated samples undergoing nitrogen annealing in a quartz boat instead of a graphite box (A2). Post-Ann(GB), shown in black, stands for a sample without irradiation undergoing graphite box annealing in nitrogen directly after exfoliation (A3). In all cases, the annealing is done at a fixed pressure of around 80 mbar without a flow. (**b**) Distribution of ZPL energies of all the observed emitters in the indicated processes.

## Results

Figure 1c–e shows the optical image and PL map with a grid of 0.5$\mu$m of representative flakes. The PL map shows the normalized integrated photoluminescence counts from 1.5 to 2.29 eV. Figure 1f–h shows spectra for several quantum emitters. We identify a quantum emitter by having a relatively sharp peak in the spectrum in the range between 1.6 and 2.3 eV. For the second-order autocorrelation measurement g^(2)^($\tau$), the spectral region containing the ZPL (grey area) is sent to the Hanbury Brown-Twiss measurement setup (see supplementary material, Sect. S4). We find clear antibunching around $\tau =0$. A luminescent emitter is regarded as quantum emitter, if g^(2)^(0) < 1 and a single photon emitter, if g^(2)^(0) < 0.5^49^. From the fits^28^ we derive the values of g^(2)^(0), as indicated in Fig.  1i-k. In supplementary material, Sect. S5, we show representative emitter spectra in different batches generated by the various treatments discussed in this work. The time traces of selected representative emitters are shown in the supplementary material, Sect. S6. We note that the lowest values of the g^(2)^(0) presented in Fig.  1i–k are not low enough for future single photon sources applications. We attribute these non-zero values of g^(2)^(0) to the strong emission background observed in our sample and that has been documented in other works using plasma irradiation and/or annealing steps for emitter activation^38–41^. This background has been attributed in other studies to residual PAHs molecules present in the environment^33^. Future works should focus on removing this background to reach close-to zero g^(2)^(0) values by developing advanced cleaning methods^33^ or to use suspended hBN flakes.

### Oxygen irradiation and carbon-rich annealing environment

In this part, we investigate the role of the irradiation process as well as the carbon presence in the post-annealing environment on the yield of luminescent emitters. In particular, we want to assess the effect of carbon-rich and carbon-poor environments on the formation yield of emitters by placing the substrates in a graphite box (GB) or quartz boat (QB) during the annealing step, respectively. Carbon residues and dangling carbon atoms can detach from the surface of the graphite box via thermal agitation and be sputtered the surface of hBN. The first batch is prepared following our standard recipe as described in the Methods section, which is schematically represented in Fig.  1b. The samples are irradiated with oxygen atoms at different kinetic energies, namely 120, 204, and 280 eV, and placed in the carbon-rich environment of a graphite box during the post-annealing process (A1: Irr + Post-Ann(GB)). The second batch is prepared identically to the first batch, the difference is that the samples are placed in the carbon-poor environment of a quartz boat during the annealing process (A2: Irr + Post-Ann(QB)). We also prepare a third batch, the reference batch, that is not irradiated and directly post-annealed in the graphite box (A3: Post-Ann(GB)). All post-annealings for all batches are done with the same annealing conditions as those described for the standard process.

In Fig.  2a, the emitter density is plotted along the irradiation energy for the three different processes described, and the emitter density is listed in Table 1. In the case of no oxygen irradiation (A3), we observe an emitter density of 0.004±0.002 $\#/\mu m^2$. The first batch (A1), undergoing the standard process (shown in purple), shows higher emitter densities of 0.018±0.003, 0.019±0.002, and 0.009±0.001 $\#/\mu m^2$ for irradiation at kinetic energies of 120 eV, 204 eV, and 280 eV, respectively. The highest yield is obtained with 204 eV and shows that a combination of oxygen irradiation and post-annealing generates a larger amount of emitters than a simple activation with annealing only. This is in line with our previous study ^28^, which discussed the fact that the oxygen irradiation amorphizes the top few layers and the subsequent inert annealing recrystallizes the layers, leading to the possible formation of optically active defects. The amorphization of the top layer is supported by previous Molecular Dynamics calculations^28^. Furthermore, Raman measurements performed on our samples after irradiation (supplementary material, Sect. S2) show a broadening of the hBN $\textrm{E}_{2g}$ peak which is a hallmark of the formation of cystallographic defects in hBN^50^. Additionally, Raman measurements after annealing in GB indicate that amorphous carbon has been deposited at the surface of hBN (see supplementary material section Sect. S2). This observation supports our hypothesis that the GB introduces carbon during the annealing process.

We observe a similar trend in the emitter density versus irradiation energy in the second batch (A2), shown in an orange curve. However, the density of emitters is significantly lower for all the irradiation energies compared to the first batch (A1). This difference of approximately a factor of 2 reveals a clear significance of the presence of carbon in the annealing environment.

Figure 2b shows that the distribution of ZPL energies is much broader for post-annealing in a carbon-rich environment, indicating emitters with different microscopic structures. This could have several causes. A large number of carbon related color centers in the hBN lattice has been proposed, (V_B_C_N_^–^, C_2_C_B_, C_2_C_N_, C_B_C_N_C_B_C_N_^19,27,29^), which all have different ZPL energies. Alternate explanations include the presence of PAH molecules^33^ pinned to the defect sites on the hBN^42^.

**Fig. 3:**
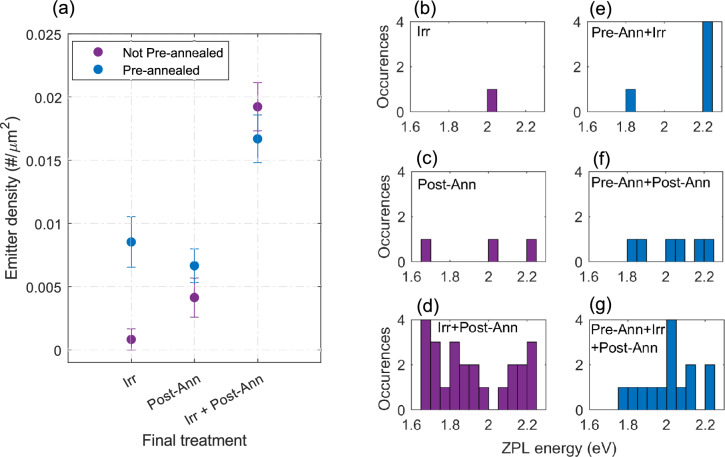
(**a**) Comparison of emitter densities in samples with and without pre-annealing in Type I hBN samples. Blue markers indicate the samples that have undergone pre-annealing treatment, and the purple ones indicate those that have not. The x-axis represents the final treatment undergone by the sample. Panels (**b**)–(**g**) show the ZPL energy distribution for the samples indicated in each plot. Note here that all indicated irradiation is done at $\sim$ 204 eV, and all post-annealing was carried out in a graphite box

### Oxygen pre-annealing

In this section, we discuss the influence of pre-annealing in a molecular oxygen environment applied prior to irradiation and post-annealing processes. This pre-annealing step aims to remove any organic molecules on and around the hBN surface that might create emitters. This cautious approach is inspired by the work of Neumann et al.^33^, which reported completely dark emission in such flakes after inert post-annealing, suggesting a correlation between organic residues coming from the micromechanical exfoliation of hBN crystals and emitter formation.

All the samples presented in this section undergo a pre-annealing in an environment of 10% oxygen in an argon atmosphere at 700°C for 2 h prior to any other treatments (see Methods). The different batches of samples that we discuss in this section are: sample before any further treatment, i.e., pre-annealing only (B1: Pre-Ann), irradiated with oxygen atoms without post-annealing (B2: Pre-Ann + Irr), not irradiated but post-annealed (B3: Pre-Ann + Post-Ann(GB)), and irradiated followed by post-annealing (B4: Pre-Ann + Irr + Post-Ann(GB)). It is important to note that batch B4 is the same as the standard process batch A1, but first has undergone the pre-annealing treatment. All irradiation in this section is done at $\sim$204 eV, corresponding to the maximum yield of emitters as shown in Fig.  2a, and all post-annealing is done with the same parameters as in the standard process (A1).

We observed a low emitter density of 0.009±0.002 $\#/\mu m^2$ in flakes undergoing oxygen pre-annealing alone (B1), with some exhibiting single-photon behavior (Fig.  1f, i). Figure 3a and b–g illustrate the influence of oxygen pre-annealing, comparing emitter densities and ZPL energy distribution for different treatment combinations. Our results show that in batch B4, where the standard process (Irr+post-Ann) is performed on pre-annealed samples, a higher emitter density of 0.017 ± 0.002 $\#/\mu m^2$ is generated. This density is similar to the emitter density produced by the standard process without pre-annealing (A1), as shown in the previous section.

**Fig. 4:**
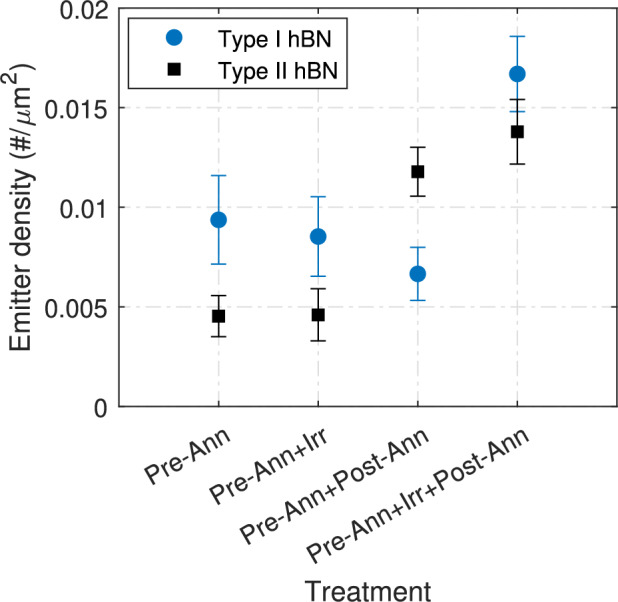
The density evolution of emitters on pre-annealing only (**B1**), pre-annealing followed by irradiation (**B2**), pre-annealing followed by post-annealing in nitrogen in graphite box (**B3**), pre-annealing followed by irradiation and post-annealing (**B4**). The blue circles are the results from Type I hBN flakes, and the black squares are those from Type II hBN flakes.

We find some differences in our methods and measurement results compared to those reported by Neumann et al.^33^, the most notable being the observation of a non-zero emitter density in pre-annealed samples. There is a possibility that high-temperature annealing in an oxygen atmosphere activates some color centers, as shown in previous works^10,51,52^. This observation could also be caused by residual organic molecules from solvents or tape residue that cannot be removed by oxygen annealing alone. Molecules beneath the flake are shielded from oxygen gas, and the heat may cause them to form fluorescent PAHs^33^. However, we observe a significant increase in emitter density after irradiation, suggesting that crystallographic defects play an important role in emitter formation. Some emitters found after the standard process may result from molecular emitters formed during fabrication steps, which may not be entirely removed even after undertaking an oxygen pre-annealing step. Nonetheless, the consistently higher emitter density observed with our standard process suggests an important role of crystallographic defects in the generation of emitters in hBN. It is well known that crystallographic defects can act as emitters (“color centers”) themselves^53,54^. Another possibility is that they act as pinning sites^55,56^ for luminescent molecules, where the pinning may or may not affect the emission properties of the molecule^34–36,42^. In the future, incorporating additional cleaning with solvents that can eliminate molecular emitters without leaving their own residues, similar to the method used by Neumann et al.^33^, could help to rule out the possibility of molecules beneath the hBN flake or pinned on surface defects induced by irradiation.

### Comparison between different hBN types

To study the role of impurities in the hBN samples, we conducted similar experiments (B1, B2, B3, B4 in Table 1). We compared hBN from HQGraphene (referred to as Type I hBN) and crystals synthesized in a high-pressure, high-temperature process^47,48^ (referred to as Type II hBN) as shown in Fig.  4. Type II hBN is of higher crystallographic quality than Type I hBN and should contain fewer carbon atoms^48,50,57^. Without applying the standard process of generating quantum emitters, we find that Type I hBN has a twice larger emitter density than Type II hBN. However, when the standard process of irradiation and post-annealing is applied, we find similar emitter densities of 0.014±0.002 #/$\mu m^2$, with that of Type I hBN being slightly higher.

The low impurity concentration of Type II hBN aligns well with the standard scenario of defect formation discussed earlier. This observation can be linked to the contribution of optically active defect centers (”color centers”). At the same time, it may also suggest the possibility of a molecular origin supported by the presence of defects. Low-quality hBN flakes, due to their low crystallinity, may have more cracks or edges where molecular species can integrate. There is a higher chance that the organic molecules from the exfoliation tape or from any fabrication step remain in these regions. Consequently, the likelihood of molecular emitter formation would also be higher in these low-crystalline flakes because oxygen preannealing removes many but perhaps not all molecular emitters.

## Conclusion and discussion

In summary, we have shown the influence of different fabrication methods on the formation of quantum emitters in hBN. We conclude that (i) oxygen irradiation has a strong influence on the emitter density, with an optimal kinetic energy of 204 eV. Additional Raman measurements on our samples indicate that a larger number of defects are generated after irradiation (see supplementary material Sect. S2), we can conclude that the irradiation step induces defects at the surface of hBN. This point is also supported by previous molecular dynamics calculations^28^. (ii) Post-annealing in the graphite box generates an increased emitter density by approximately a factor of 2 as compared to that in the quartz boat, with a wider distribution of zero-phonon lines toward the (infra)-red. Raman measurements on the same samples after annealing shows the appearance of a new peak around 1600 $\textrm{cm}^{-1}$ which is consistent with the presence of carbon^13^ deposited by the graphite box. These observations support the crucial role played by carbon in the formation mechanism of the quantum emitters in hBN. (iii) Oxygen pre-annealing does not have a strong influence on the emitter density, with the density being almost the same for the flakes treated by the standard process. This point indicates that molecular residues adsorbed at the surface of hBN do not seem to play a crucial role in the formation mechanism of the measured emitters. However, we cannot rule out the possibility that molecules situated in cracks and under the sample can be protected from the oxidizing atmosphere of the preannealing step and thereby play a role in the formation of quantum emitters. (iv) hBN from HQGraphene shows a slightly higher emitter density compared to the more pristine NIMS hBN. This observation also points to the preponderant role of crystallographic defects in the formation of quantum emitters in hBN since NIMS hBN is known to host a lower density of intrinsic defects than HQGraphene^50^. In general, these observations indicate the crucial role cystallographic defects play in the formation of quantum emitters in hBN.

Now, turning from conclusions based on our data to a discussion of related open questions, we note that crystallographic defects do not always have to be the emitters themselves, as defects may act as pinning sites that attract organic molecules from the environment^33,42,55^. Molecules attaching to edges^56^ and undercoordinated sites^55^ in 2D materials have been reported in the literature and could also explain our observations. Recent calculations have shown that defects in hBN can pin down and stabilize PAH molecules while leaving their quantum optical properties almost untouched^42^. We therefore think that this is a valid hypothesis that needs to be investigated in future experimental works. Measurements on monolayer hBN involving scanning transmission electron microscope and electron energy-loss spectroscopy could be used to clarify the true nature of the quantum emitters with emission around 2 eV in hBN, a topic of still ongoing debate^31^. Another point worth noting is that the density of emitters created with our method is generally quite low, which seems to be a common feature in hBN samples treated by low-energy or plasma irradiation followed by subsequent annealing^40^. Understanding their true microscopic nature and their formation mechanism in detail will also help optimize the density of emitters created through these ubiquitously used activation methods.

Future work should focus on distinguishing the contributions of intrinsic crystallographic defects from molecular quantum emitters. Advanced cleaning techniques^33^ combined with new spectroscopic methods able to measure Raman signals at the single-molecule level^58^ and STEM imaging could clarify the nature of 2 eV emitters in hBN. Identifying their origin is essential for developing targeted generation methods and controlled fabrication, advancing quantum technologies. Our systematic analysis of fabrication parameters provides a foundation for this effort.

## Supplementary Information

Supplementary Information.

## Data Availability

The datasets generated during and/or analysed during the current study are included in the Supplementary Information files or are available from the corresponding author on reasonable request.
