# Supplementary material for: Systematic investigation of the generation of luminescent emitters in hBN via irradiation engineering
Source: Sci Rep. 2025 Nov 17;15:40288. doi: 10.1038/s41598-025-24064-x (PMC12623796; doi:10.1038/s41598-025-24064-x)
Supplement: Supplementary file 1 — Supplementary Information. [file 41598_2025_24064_MOESM1_ESM.pdf]

Supplementary information for: Systematic investigation of the generation of luminescent emitters in hBN via irradiation engineering

Pooja C Sindhuraj[1], José M Caridad[2,3], Corné Koks[1,4], Moritz Fischer[1], Denys I Miakota[1], Juan A Delgado-Notario [2,3], Kenji Watanabe[5], Takashi Taniguchi[6], Stela Canulescu[1], Sanshui Xiao[1,4], Martijn Wubs[1,4], and Nicolas Stenger[1,4]

[1] Department of Electrical and Photonics Engineering, Technical University of Denmark, 2800 Kgs. Lyngby, Denmark

[2] Departamento de Física Aplicada, Universidad de Salamanca, Salamanca 37008, Spain

[3] Unidad de Excelencia en Luz y Materia Estructuradas (LUMES), Universidad de Salamanca, Salamanca 37008, Spain

[4] NanoPhoton – Center for Nanophotonics, Technical University of Denmark, 2800 Kgs. Lyngby, Denmark

[5] Research Center for Electronic and Optical Materials, National Institute for Materials Science, 1-1 Namiki, Tsukuba 305-0044, Japan

[6] Research Center for Materials Nanoarchitectonics, National Institute for Materials Science, 1-1 Namiki, Tsukuba 305-0044, Japan

E-mail: niste@dtu.dk

S1. Estimation of the ion current density

The RIE reactor is driven by a high-frequency generator operating at 13.56 MHz capacitively coupled to the bottom electrode and the chamber has a circular parallel plate geometry with a plate diameter of 33.5 cm (plate area A $\approx$ 882 cm^2), enabling thus the calculation of the power density of each process. In particular, using the plate area A, the power P and the measured DC bias V_{bias}, one can give a first-order estimate of relevant parameters such as the ion current density as well as others like the fluence. We note that these are upper-bound approximations, as they assume that the RF power is entirely transferred to ion acceleration. As an example, we estimate the ion current density J for a process with power P= 20W (power density $\approx$ $22,7$ mW/cm^2) and measured DC bias, V_{bias} of 120V, taking into account the following relation between P, V_{bias}, A and J (see ref. [1]): $P = JAV_{bias}$, so $J \sim 1.9 * 10^{-4}$ A/cm2. Assuming an-ion charge $q = 1.6 * 10^{-19}$ C in the process [2], the estimated fluence F for a process duration of t=10 seconds process would then be $F = Jt/q \approx 1.2 * 10^{16}$ ions/cm^2. We finally note that the estimated fluence for such a process is consistent with the one reported by Fischer, et al. [2], considering that capacitively coupled plasma reactors typically have power transfer efficiencies of 50% or less [3, 4].

S2. Raman signals from hBN undergoing irradiation and post-annealing treatment

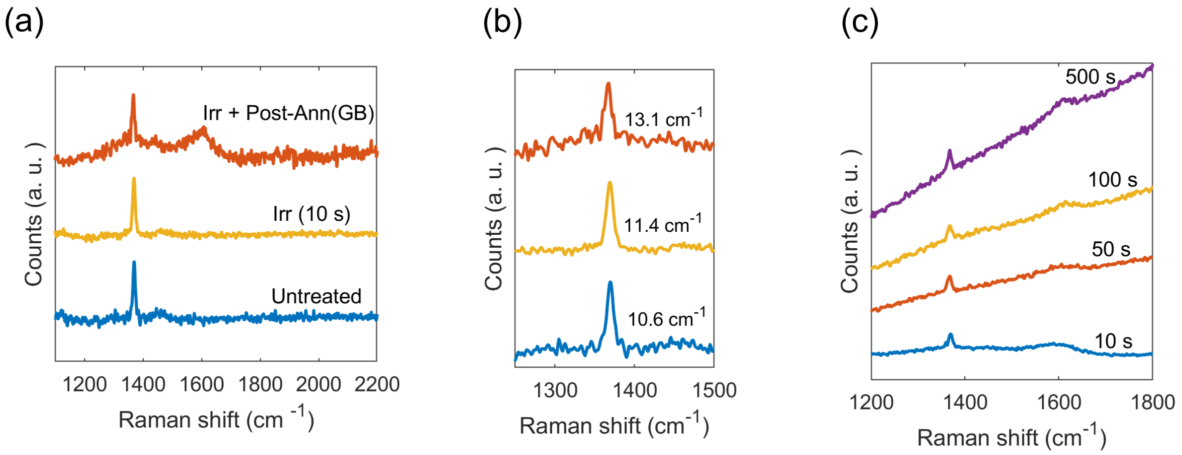

Fig. S1: (a) Raman signal from an exfoliated hBN flake (untreated), oxygen irradiated for 10 seconds (Irr (10s)), and subsequently annealed in a graphite box (Irr+Post-Ann(GB)). The band around 1600 cm^{-1} arises from carbon in the sample[5], possibly during the annealing in the carbon environment. (b) The zoom-in of E_{2g} Raman mode of hBN with the FWHM value of each peak, obtained from fitting with a Gaussian. The E_{2g} peak values for untreated, irradiated, and irradiated + post-annealed flakes are obtained as 1369.7, 1369.2, 1366.7 cm^{-1}, respectively. The increase in FWHM value is an indicator of damage in the lattice during each stage, or intuitively the formation of defects as discussed in Reference [6]. (c) Damage caused by irradiation on hBN flakes with time as evidenced from the Raman signals. The signals are measured from samples undergoing irradiation followed by post-annealing in a graphite box, with an irradiation duration as indicated for each spectrum. A longer duration of irradiation causes a huge background luminescence. The blue spectra in (c) correspond to the orange in (a) and (b).

S3. Area number density calculation

For all the emitter statistics presented, we used emitter density per area of the flakes, similar to the calculation in Reference [2]. The geometrical area of the flakes is calculated from the sample's optical image. An error of 5 μm^2 is assigned for determining the flake area. We define the areal number density,

$$D = \frac{N}{A}, \tag{1}$$

where N is the total number of emitters in 'n'number of flakes and 'A'is their total area.

The error in determining the density is given by,

$$\Delta D = \frac{\Delta N}{A} + \frac{N.\Delta A}{A^2}, \tag{2}$$

where

$$\Delta A = n.5 \quad \mu m^2 \tag{3}$$

and

$$\Delta N = \begin{cases} 1, & \text{if } N < 4. \\ \text{round(N/10)}, & \text{if } N \geq 5. \end{cases} \tag{4}$$

The function round(x) rounds off x to the nearest integer.

The following tables show all details of the flake area and emitters, which are the basis of our statistical study.

TABLE. S1: Data for emitter density calculation in Type I samples undergoing the standard process (Batch A1), for the indicated irradiation powers, and the non-irradiated sample undergoing direct post-annealing in graphite box (Batch A3) discussed in the Main text.

Irradiation Power (W)	Irradiation energy (eV)	Flake area(μm^2)	Flakes	Emitters
20	120 ±5	893.36	4	16
40	204±5	1247.84	4	24
60	280±5	1469	5	13
Non-irradiated (Batch A3)	0	726.36	6	3

TABLE. S2: Data for emitter density calculation in Type I samples undergoing the irradiation followed by post-annealing in a quartz boat (Batch A2) discussed in the Main text, for the indicated irradiation powers.

Irradiation Power (W)	Irradiation energy (eV)	Flake area(μm^2)	Flakes	Emitters
20	133 ±5	612.21	5	5
40	204±5	566.63	5	6
60	289±5	574.7	5	3

TABLE. S3: Data for emitter density calculation in Type I samples undergoing the indicated treatments as discussed in Table 1 in the Main text.

Batch	Treatment	Flake area(μm^2)	Flakes	Emitters
B1	Pre-Ann only	533.69	4	5
B2	Pre-Ann + Irr	586.1	4	5
B3	Pre-Ann + Post-Ann	901.53	6	6
B4	Pre-Ann + Irr + Post-Ann	838.62	7	14

TABLE. S4: Data for emitter density calculation in Type II hBN samples undergoing the indicated treatments as discussed in Table 1 in the Main text.

Batch	Treatment	Flake area(μm^2)	Flakes	Emitters
B1	Pre-Ann	1103.05	6	5
B2	Pre-Ann + Irr	870.23	6	4
B3	Pre-Ann + Post-Ann	1103.05	6	13
B4	Pre-Ann + Irr + Post-Ann	870.23	6	12

S4. Spectral window selection for HBT measurements

By adjusting the grating angle in our spectrometer, the spectral range of light incident on the center of the electron-multiplying charge-coupled device (EMCCD) camera, referred to as the spectrometer wavelength in the main text, can be selected. A flip mirror positioned in this optical path reflects the signal toward the Hanbury Brown–Twiss (HBT) setup. When the spectrometer wavelength is set to match the zero-phonon line (ZPL) peak, the corresponding light is directed onto the flip mirror such that its reflected path aligns with the center of the output slit. This output slit, located between the flip mirror and the HBT setup, defines the spectral bandwidth of the signal transmitted for correlation measurements.

S5. More representative emitter spectra

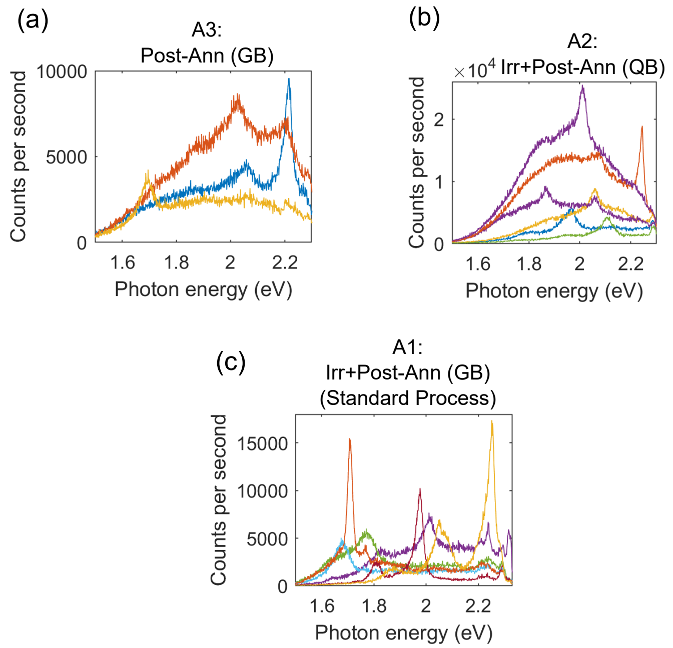

Fig. S2: Representative emitter spectra of emitters generated in (a) Batch A3, the one in which flakes are directly post-annealed in graphite box (Post-Ann(GB)) (b) Batch A2, which is irradiated followed by post-annealing in a quartz boat (Irr+Post-Ann(QB)) (c) Batch A1 undergoing the standard process, which is irradiation followed by post-annealing in a graphite box (Irr+Post-Ann(GB)). All mentioned irradiation is done at ∼204 eV.

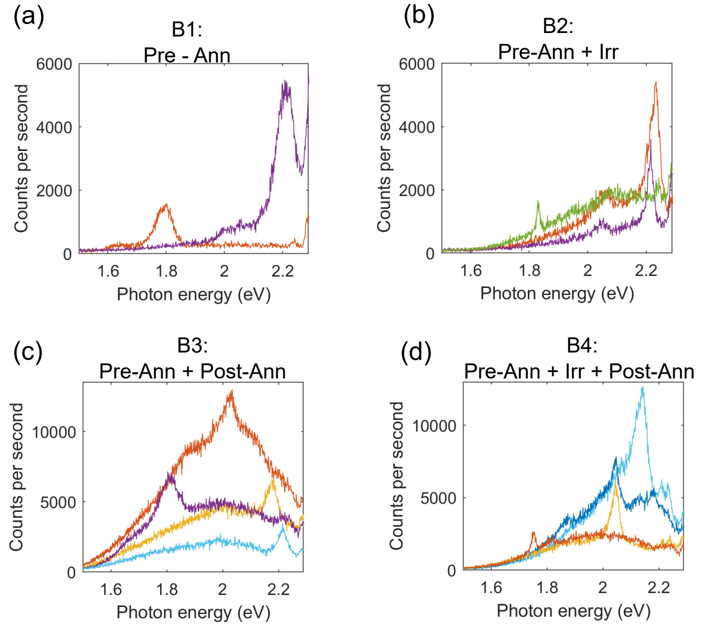

Fig. S3: Representative spectra of emitters generated in batches undergoing the pre-annealing process (a) Batch B1 with only the pre-annealing process. (b) Batch B2 undergoing subsequent irradiation. (c) Batch B3 undergoing the post-annealing treatment without an irradiation. (d) Batch B4 undergoing the standard process of irradiation followed by post-annealing. All mentioned irradiation is done at $\sim$204 eV, and all mentioned post-annealing is done in the graphite box.

S6. Photophysics and stability of selected emitters

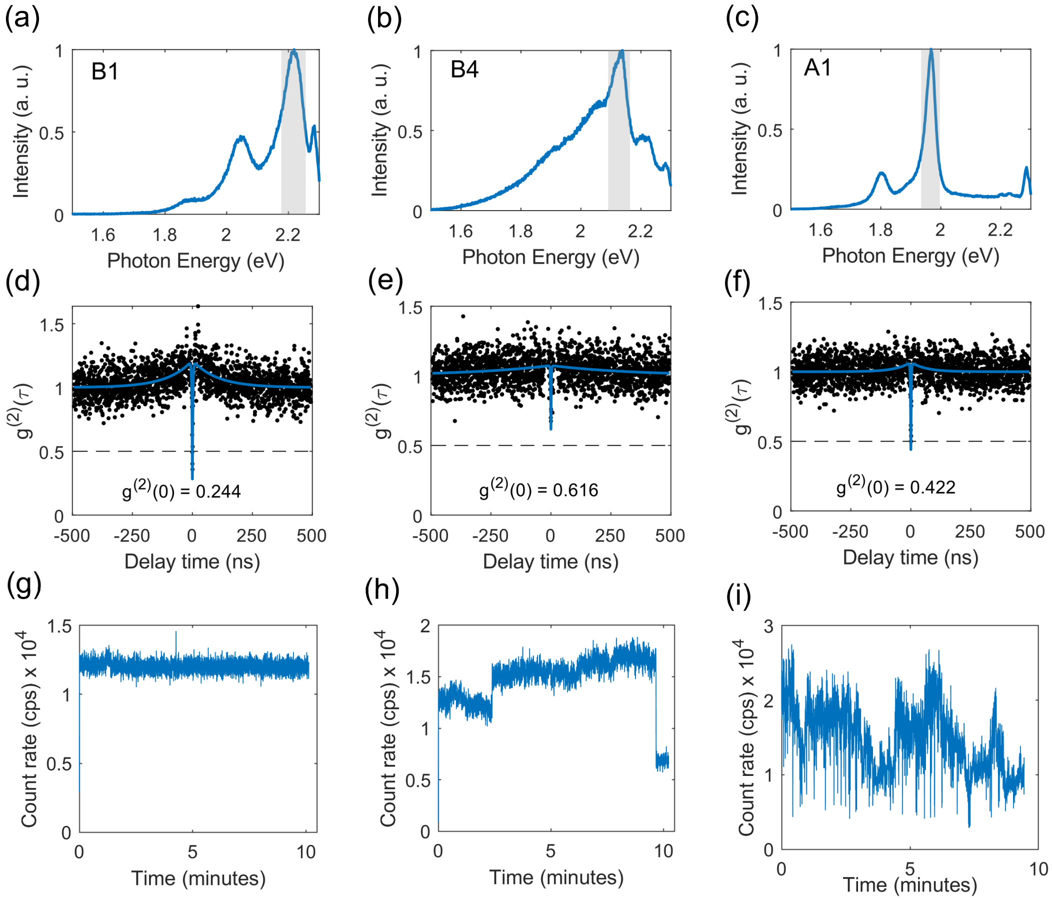

Fig. S4: Photoluminescence spectrum of a representative emitter generated by (a) pre-annealing alone (B1), (b) pre-annealed followed by standard process (B4), (c) the standard process (A1). (d-f) $g^{(2)}$ function of the emitters shown in (a-c) from the spectral region shaded in grey. (g-i) Time trace of the corresponding emitters shown in (a-c) on continuous excitation over a period of 10 minutes.

References

[1] Michael A Lieberman and Allan J Lichtenberg. *Principles of Plasma Discharges and Materials Processing.* John Wiley & Sons, 2024.

[2] M. Fischer et al. "Controlled generation of luminescent centers in hexagonal boron nitride by irradiation engineering". In: *Science Advances* 7.8 (Feb. 2021), pp. 7138–7155. ISSN: 2375-2548. DOI: 10.1126/sciadv.abe7138. URL: https://www.science.org/doi/10.1126/sciadv.abe7138.

[3] M Mohamed Salem, J-F Loiseau, and B Held. "Impedance matching for optimization of power transfer in a capacitively excited RF plasma reactor". In: *The European Physical Journal-Applied Physics* 3.1 (1998), pp. 91–95.

[4] Jiamao Gao et al. "Self-consistent simulation of the impedance matching network for single frequency capacitively coupled plasma". In: *Journal of Physics D: Applied Physics* 55.16 (2022), p. 165201.

[5] Songyan Hou et al. "Localized emission from laser-irradiated defects in 2D hexagonal boron nitride". In: *2D Materials* 5.1 (Jan. 2018), p. 015010. ISSN: 20531583. DOI: 10.1088/2053-1583/aa8e61.

[6] Léonard Schué et al. "Characterization methods dedicated to nanometer-thick hBN layers". In: *2D Materials* 4.1 (Mar. 2017), p. 015028. ISSN: 20531583. DOI: 10.1088/2053-1583/4/1/015028.
